# Older cancer patients' information and support needs surrounding treatment: An evaluation through the eyes of patients, relatives and professionals

**DOI:** 10.1186/1472-6955-8-1

**Published:** 2009-01-19

**Authors:** Elise R Posma, Julia CM van Weert, Jesse Jansen, Jozien M Bensing

**Affiliations:** 1 Netherlands institute for health services research (NIVEL), P.O. Box 1568, 3500 BN Utrecht, the Netherlands; 2 Department of Health Psychology, Utrecht University, the Netherlands; 3 Amsterdam School of Communications Research (ASCoR), University of Amsterdam, Kloveniersburgwal 48, 1012 CX Amsterdam, the Netherlands

## Abstract

**Background:**

Providing cancer patients with adequate treatment information is important for patients' health, well-being and satisfaction. Nurses play an important role in patient education. So far, few studies focused on the specific information needs of older cancer patients surrounding chemotherapy treatment. Given the growing incidence of cancer among older individuals, insight in these needs is crucial. This article describes the views of older cancer patients, their relatives and professionals on older patients' specific communication needs regarding chemotherapy treatment.

**Methods:**

A qualitative design was used. Five focus group interviews were held with older cancer patients and their partners (two groups) and professionals with a background in nursing, oncology, gerontology and/or patient-provider communication (three groups). In addition, face to face in-depth interviews were conducted with older cancer patients. A total number of 38 patients and relatives participated, with a mean age of 67.6 years. The focus groups and interviews were audio-recorded for subsequent transcription and analysis.

**Results:**

Older people have more difficulties processing and remembering information than younger ones. A trustful environment appears to be a prerequisite for reflection of older patients on the information provided and individualized information is essential to enhance memory of information. However, the results show that both patients and professionals experienced insufficient exploration of the patients' personal situation and individual information needs. Patients also strengthened the importance of sensitive communication, e.g. showing empathy en emotional support, throughout the continuum of cancer care. Moreover, potential areas of improvement were identified, including engaging the patients' relatives and encouraging patients and relatives to ask questions.

**Conclusion:**

Patient education should be more tailored to older cancer patients' individual information and support needs and abilities by exploring the required amount and content of information, treatment goals and expectations. Nurses can establish a trustful environment by showing empathy and emotional support. Recommendations are given to enhance recall of information in older patients; information giving should be more structured by summarizing and repeating the most important, personally relevant information. To adapt to specific information needs, communication training for nurses and the use of aids such as a question prompt sheet could be useful tools.

## Background

Cancer is frequently a disease of older individuals, and is the second leading cause of death in the United States for those aged 65 and older [1]. More than 55% of the patients newly diagnosed with cancer are 65 years or older [2]. Providing patients with adequate information, advice and support around treatment is an important component of care, in which nurses play an important role. The benefits of good patient education for cancer patients may include greater satisfaction with treatment choices, improved ability to cope during the diagnosis, treatment, and post-treatment phases, and reductions in anxiety and mood disturbances [3-5]. Given the growing incidence of older individuals and the importance of providing adequate information, this article aims to achieve a better understanding of information needs of older cancer patients surrounding an invasive treatment such as chemotherapy.

The information needs of cancer patients vary considerably across individuals [6-8]. Patients desire different types and amounts of information depending on their type of cancer, the extent of disease progression, and their unique personal life circumstances [6]. Personal relevance determines for a large part whether information will receive attention by a patient or not. Personal relevant information is processed more deeply (e.g. receives increased attention), leading to better comprehension, memory storage and use of the information [9,10]. Thus, an indirect effect of tailoring information to the individual patient may be enhancing patients' memory. Recall of information from a medical consultation is important for patients' health and well-being, as it has been associated with decision making [11], good adherence to recommended treatment [11,12] and patient satisfaction [12,13]. However, patients forget much of the information provided [6,7].

**Figure 1:**
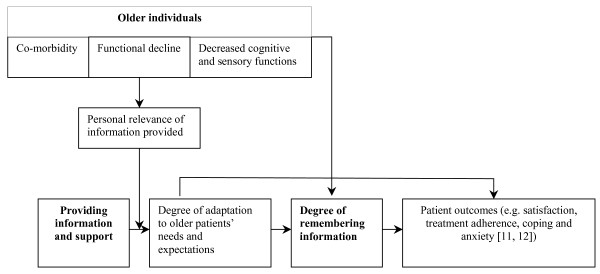
**Conceptual model of the relationship between 'patients needs' and 'recall of information' among elderly patients**.

The relationship between personal relevance of information provided, tailoring the information to patients' needs, the extent to which information is remembered and patients outcomes has been visualised in Figure 1, showing the conceptual model underlying this study. The expected role of specific characteristics of older cancer patients will be explained below.

Older individuals' information needs might differ from the needs of younger patients, since their perceptions of cancer and treatment seem to differ from younger patients, as well as their knowledge of the disease [14]. In addition, older cancer patients are likely to have concurrent diseases (co-morbidity). The prevalence of functional impairments is also higher among older cancer patients. Functional decline refers to the ability of the individual to perform activities of daily living (ADL), such as using the toilet, dressing and eating, and to perform instrumental activities of daily living (IADL), such as using transportation, shopping, taking medication, and preparing meals [15]. There is evidence that functional status has a bigger impact than co-morbidity on treatment decisions [16] and how well patients do after treatment in terms of complications and length of hospital stay [17]. As functional decline often determines the ability to return home, it is expected to affect patients' and relatives' information and support needs [18]. Especially, the potential impact of treatment on older people's ability to undertake activities of every day living may be important in terms of what sort of information they want. To add, remembering medical information and treatment recommendations might be a bigger problem for older patients, as cognitive, vision and hearing functions decrease with age [12,14]. Besides, older individuals have more difficulty in organizing and storing information [11], which has a negative influence on recall of information.

Thus, it is crucial to gain insight in the information and support needs of older individuals, to tailor patient education about cancer treatment according to these needs, enhance recall of information and, ultimately, improve other patient outcomes such as satisfaction, medical compliance, coping with illness and well being.

Numerous studies have evaluated cancer patients' information needs and suggested that the vast majority of cancer patients want as much information as possible whether it is good or bad [19,20]. However, a recent systematic literature review revealed that hardly any studies investigated the specific needs of older cancer patients surrounding treatment [21]. The authors therefore included studies in the review in which some of the patients were older (i.e. 65 years or older) and the presence or absence of age differences were reported. They identified 17 studies that met their inclusion criteria, the results of which suggest that the majority of older cancer patients want to receive relevant information about their treatment. Yet, the results also indicate that although older patients prefer to receive information about the most important aspects of the disease and treatment, they are relatively less interested in extensive and detailed information [21].

This article describes the views of both older cancer patients and professionals, on patients' specific information needs preceding chemotherapy. The study is part of a larger, prospective study that aims to improve patient education for older cancer patients during nursing consultations preceding chemotherapy. To obtain insight in the views of older cancer patients and professionals, the following research questions were addressed:

1. Which differences between older and younger patients should be taken into account by nurses during patient education?

2. What are the information and support needs of older cancer patients facing chemotherapy treatment?

3. How can older cancer patients' recall of information be enhanced by nurses?

## Methods

### Design

The present study employed a qualitative design using five focus group interviews and five face to face interviews. A semi-structured interview, which consisted of open-ended questions, was used to explore the opinions of cancer patients and professionals concerning the specific needs of cancer patients surrounding chemotherapy treatment. The overarching prospective study, including the present study, was approved by The Medical Ethical Committee of the University Medical Centre Utrecht (VOICE 04/184), supplemented by local feasibility statements from the participating hospitals. Eligible patients were sent or given a letter by the hospital nurse, explaining the study aims and demands as well as explaining that anonymity was guaranteed and refusing to participate would not influence their treatment. After a few days, patients were phoned by one of the researchers to answer their existing questions about the study. Patients who agreed to be interviewed were visited at home, except for one patient who preferred to be interviewed at the hospital. They signed written informed consent prior to the interview. Focus group patients came to the research institute, which is located in the centre of the Netherlands. Personal information (e.g. name and address) was securely stored separately from the data collected in the interviews to make sure that the participants were not identifiable.

### Setting

In general, patient education about chemotherapy treatment in Dutch hospitals is provided by (specialized) oncology nurses. During a consultation which lasts approximately one hour, information is provided about the treatment, side effects and how to deal with side effects. To explain issues concerning chemotherapy treatment, a booklet 'Treatment Guide Chemotherapy' [22] is often used in the Netherlands during these consultations. This booklet contains an overview of the most important treatment issues and is given to patients after (or sometimes before) the consultation. The consultation usually takes place two weeks to one day before the first chemotherapy treatment starts. No clear difference exists between patient education for younger and older patients.

### Subjects

**Figure 2:**
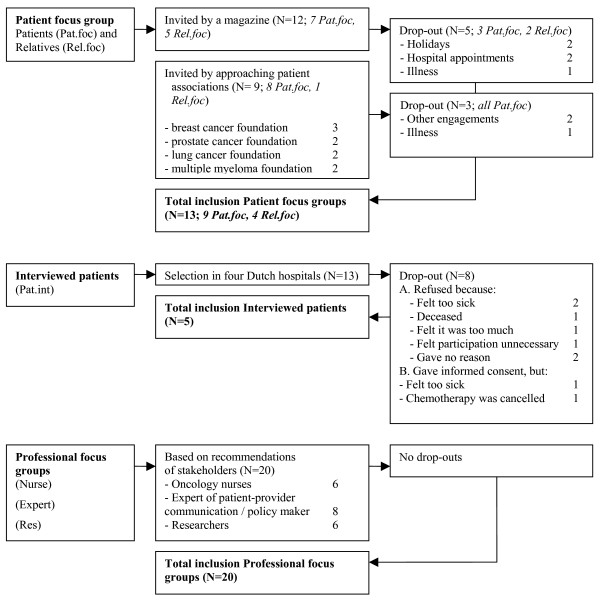
**Flow chart of the study**.

Five focus group interviews and five one-on-one interviews were conducted with a total number of 38 subjects. Two focus groups consisted of older cancer patients and their partners who had received a treatment like chemotherapy in the past; three focus groups consisted of professionals with a background in oncology, gerontology, nursing care and/or patient-provider communication. For the interviews, older cancer patients (≥ 65 years) were included who had just finished their second chemotherapy treatment to obtain retrospective information about their needs surrounding treatment as well as their experiences regarding the fulfilment of these needs. The focus group patients and their relatives were selected by an advertisement in a magazine (called "Plus Magazine") for older individuals (first patient focus group) and by approaching seven patient associations (second patient focus group). Twelve people (seven patients and five partners) responded to the advertisement. Seven of them (four patients and three partners) attended the meeting. The other five were unable to come due to holidays (n = 2), hospital appointments (n = 2) and illness (n = 1). In addition, there were nine enlistments (eight patients and one partner) of four patient associations: breast cancer foundation (n = 3), prostate cancer foundation (n = 2), lung cancer foundation (n = 2) and multiple myeloma foundation (n = 2). Six of them (five patients and one partner) attended the meeting. Three cancelled due to other engagements (n = 2) and illness (n = 1). Figure 2 provides an overview of the selection of subjects and the drop-out. The abbreviations of the participants groups mentioned in the flow chart correspond with the abbreviations in de result section (see also 'Data analysis').

Interviewees were recruited in four Dutch Hospitals. To be eligible for the interview, patients had to meet the following inclusion criteria: 1) aged 65 years or older, 2) receiving chemotherapy for the first time or for the first time in 5 years, 3) sufficient command of the Dutch language and 4) no history of cognitive deficiencies according to the medical file. A sample of 13 consecutive participants was selected at the time of their second chemotherapy treatment. Of these, five subjects participated in the study.

Figure 2 gives an overview of the drop-outs. Six refused to participate because two felt too sick, one deceased, one felt it was too much, one felt participation was unnecessary and one refused without giving a reason. Of the seven patients who gave informed consent, two could not be included in the study: one was too sick and the other patients' chemotherapy treatment was cancelled, leaving five patients. Socio-demographic data, disease characteristics and treatment characteristics were also collected. Table 1 provides an overview of the patients' characteristics.

**Table 1:** Characteristics of patients and proxies attending the focus groups (n = 18)

	Patients focus groups	Patients interviews	Proxies	Total
**Gender**				
Male	6	2	1	9
Female	3	3	3	9
				18
**Age **(SD)	66.67 (5.31)	70.40 (2.79)	66.25 (5.91)	67.61 (4.95)
**Education level**				
Low	3	5	2	10
Middle	3	-	-	3
High	3	-	2	5
**Diagnosis**				
Breast cancer	2	-		
Prostate	2	-		
Lung cancer	1	4		
Ovary cancer	1	1		
Multiple myeloma	1	-		
Lymph gland cancer	1	-		
Colorectal cancer	1	-		
**Treatment**				
n patients (number of treatments (*M*))				
Chemotherapy	6 (10.67)	5 (2)		
Operation	7 (1.71)			
Radiotherapy	4 (28.25)			

The professionals were selected based on recommendations of stakeholders in these areas. A total number of 20 participants were divided into three professional focus groups, to cover opinions of professionals in different fields of expertise: one group of oncology nurses (n = 6), one group of policymakers and communication trainers (n = 8), and one group of researchers (n = 6). Table 2 provides an overview of the professionals' characteristics.

**Table 2:** Characteristics of professionals attending the focus groups (n = 20)

	**Area of knowledge**	**(N)**
**Researchers**	Psychologist-researcher cancer	2
	Researcher Cancer care	1
	Professor Psychiatry of elderly	1
	Researcher Cognition of elderly	1
	Psychologist and expert of educational booklets	1
	TOTAL	6
	Male	3
	Female	3
**Experts of patient-provider**	Education adviser and communication trainer	3
**communication**	Representative of cancer patient association	1
	Policymaker Care and Elderly (government)	2
	Social worker Cancer Institute	1
	Coordinator Nursing Department	1
	TOTAL	8
	Male	1
	Female	7
**(Oncology) nurses**	Oncology nurse (specialist)	3
	Oncology nurse	2
	Nurse-practitioner Oncology	1
	TOTAL	6
	Male	0
	Female	6

### Data collection

An interview schedule was designed to address the study aims. The interview schedule comprised three main sections: 1) differences between older and younger patients; 2) information and support needs and 3) recall of information. Table 3 provides an overview of the questions. The focus groups were led by experienced moderators. In semi-structured format, the participants were encouraged to express their perspectives, beliefs and experiences about the issues put forward in the questions. The sessions lasted approximately 45 minutes in the patient groups and one hour and 15 minutes in the professional groups. For the interviews, a comparable interview protocol was used. The interviews lasted approximately one hour.

**Table 3:** Interview protocol

**Topic list discussed with participants (patients and professionals)**
**Differences between older and younger patients**
a. Are there differences between older and younger patients of which nurses should take account during their patient education?
b. What can nurses do to take these differences in account?

**Needs at the beginning of treatment**
a. What are the most important educational goals when preparing patients for an invasive treatment like chemotherapy? What are the needs of older patients and their relatives?
b. How can nurses tailor education about chemotherapy to the individual needs and circumstances of older patients and their relatives?
c. Are there any other important aspects to consider during patient education about chemotherapy?

**Recall of information**
a. Which information is considered most important to remember at the beginning of the treatment?
Extra asked to professionals: Which information should be provided verbally (during consult) and which information can be provided in a written form?
b. What can nurses do to ensure that patients and their relatives will remember relevant information provided?

### Data analysis

The focus groups and interviews were audio-recorded for subsequent transcription and analysis. Although qualitative research is very useful in the exploration and description of phenomena, researchers using a qualitative design are more challenged to guarantee validity of the study. To obtain valid results and to overcome the limited perspective of an observer [23], the transcripts of the interviews were analysed independently by two observers (ERP and JCMW) and emergent themes were identified structurally. Themes are formed by unifying statements of participants about the various subjects in the interviews [24,25]. In our study, data were initially categorized according to responses to questions of the interview protocol (see Table 3). Data from each question were then examined systematically to identify particular categories of meanings following the qualitative content analysis [24]. First, meaningful units were identified (e.g. a 75 years old person does not know how many years he or she has left). Then, condensed meaning units were described, by identifying the underlying meaning of the units (e.g. older persons view their future differently from younger persons). The next phase was identifying the subtheme (e.g. older persons may have different life expectations than younger patients). Finally, a collection of different subthemes was identified as a theme (e.g. differences between older and younger patients) [24].

In the result section, quotes are illustrative and reflect the responses given by the participants. Quotes were selected on basis of the criteria that a quote should illustrate the category of meaning sufficiently, that it adds a new perspective and that the quotes represent the opinions of different participants. The participants are not personally identifiable. Numbers and letters are used to represent: patient in focus group (Pat.foc), patient in the one-on-one interview (Pat.int), relative in focus group (Rel.foc), researcher (Res), expert of patient-provider communication/policy maker (Expert) and (oncology) nurse (Nurse). There were no contradictions within the patient groups and within the professional groups, unless mentioned in the result section.

## Results

The results of this study are discussed below. The findings reflect patients', relatives' and professionals' opinions about differences between older and younger patients (research question 1), older patients' need of information and support (research question 2), and remembering information (research question 3). Table 4 provides a summary of the results.

**Table 4:** Opinions expressed in focus groups and interviews

	Patients focus groups	Patients interviews	Professionals focus groups
**Differences between older and younger patients**			
			
Individual variance, apart from age differences	-	x	x
Older patients have more difficulty processing information	x	-	x
Older patients have more life experience and different expectations about the future	x	-	x
The difference in life expectations has its impact on the decision to undergo a treatment or not	-	-	x
Older patients are more vulnerable (co morbidity) and have more resistance to ask for assistance: they need guidance in asking for and receiving help	x	-	x
			
**Needs of older cancer patients**			
			
Concrete information about their disease and treatment	x	x	x
Exploring the patient's personal situation	x	x	x
Exploring information needs of patient and relative	x	x	-
Tailor information to the patient's specific needs and situation	x	x	x
Preferences of timing of providing the information differs among patients	x	-	-
Empathy, support and reassurance, for both patient and relative	x	x	-
Exploring patient's expectations or ideas about chemotherapy treatment	-	-	x
Exploring patient's goals of undergoing treatment	x	x	x
Engage both patient and relative actively in patient education	x	-	-
Encourage patient and relative to ask questions	x	-	x
Communication training for nurses to tailor to patients' needs	x	x	x
Follow-up training, in which learning in practice is the key ingredient.	-	-	x
			
**Enhancing recall of information**			
			
Bring a (younger) person	x	x	x
Check: does patient understand the information?	x	x	x
Set priorities and offer information in a structured manner	x	-	x
Spread information over time (piece by piece)	x	-	x
Speak in clear language/avoid jargon	x	-	-
Summarize most important information	x	-	x
Combine different methods of offering information	-	x	x
Use question prompt list	x	-	x

*Note*. x = opinion is expressed by participants

### 1. Differences between older and younger patients

Two professional groups mentioned that differences between patients are the result of *individual variance*, rather than age differences.

"Apart from age influences, there is just a lot of variance [between patients ERP]." (Nurse3)

"65 or 85 years old: the actual age doesn't matter." (Res4)

These individual differences were also found in the face to face interviews, as some patients prefer very detailed information, while others prefer little information.

"I want to know everything." (Pat.int3)

"All the information and explanations don't cure. At least, that's my opinion, but every human being is different. I personally don't need it." (Pat.int1)

"I actually don't want to know what the future will bring." (Pat.int5)

#### Processing information

Despite individual differences, the participants in the patient groups and in one professional group (of experts) agreed that older patients in general have *difficulties with processing and remembering information*.

"Short term memory in older individuals is an important factor [in providing patient education ERP]." (Rel.foc1)

"Yes, you have to read information more often." (Pat.foc2)

"That's obvious, there is a difference in the way the brain functions; cognitive differences." (Expert1)

#### Life experience and expectations

Both patient and professional groups considered *life experience *the most important difference between older and younger patients. Older individuals often have other diseases to cope with, so they are more used to being ill. They are more reconciled with the idea of becoming ill than younger persons are.

"And often they already have functional problems, lung diseases for example, so they are more used to being sick." (Nurse1)

"Emotions are different for older people [..]. Young persons shouldn't get ill in any case. When you grow older, you are aware that your chances to get cancer increase." (Pat.foc5)

Consequently, according to both patient groups, interviewees and professional groups, older persons view their future differently from younger persons and are better able to cope with illness than younger ones.

"I think that when you grow older, you're able to handle situations better. When you are 30 years old, you'll have a whole future in front of you. But when you are over 70 years old, the largest part of your life lies behind you. Also, when you are being treated for cancer, the chance that you will die of disease other than cancer grows." (Pat.foc7)

"Well, I'm 75 years old and I don't know how many years there are left." (Pat.int3)

#### Accepting help from others

Older patients may suffer from co morbidity, which can complicate treatment and influence their view of the disease. However, although more vulnerable, older patients may not ask for help when necessary and often need *guidance in asking for and receiving help*, according to both patient and professional groups.

"Nurses should realize that [older ERP] patients might need more attention." (Pat.foc5)

"For older individuals, it's difficult to ask for help. That is something I hear very often. [..] Older people frequently say: 'But the children, they are busy working, they don't have the time to help'. So to ask a neighbor for help, they have to overcome their resistance. [..] And you should stimulate them, involve them, for example: 'Your daughter actually appreciates it when you ask for help; they often can do so little to help'. The family is often powerless to do anything, because mum or dad keeps the door closed." (Nurse3)

### 2. Needs of older cancer patients

Both patient groups, interviewees and professional groups stated that patients in the first place want to receive *concrete information about their disease and treatment*, like diagnosis, prognosis, side effects of the treatment, complications, and practical information.

"I think the most important goal is to provide information about expected side effects of the treatment." (Expert8)

"The nurse should explain something about possible infections you can get after the chemotherapy treatment." (Pat.foc9)

"The medical [explanation ERP] was most important, off course." (Pat.int1)

#### Exploring patients' needs and tailoring information

The goal of patient education should further depend on individual preferences. Thus, directly *asking patients about their (information) needs and tailoring the information *to these specific needs is considered very important. In both patient and professional groups it was rendered important to ask patients what type information they want to receive.

"It's important to tailor patient education to the patients' personal information needs. That's a basic skill nurses should have." (Pat.foc5)

"Ask them! Just ask them what they need." (Rel.foc1)

According to a patient group and interviewees, nurses pay insufficient attention to the individual patients' information needs.

"My experience is that during patient education, no distinction was made between different patients. For example, we didn't feel like talking about getting in contact with other patients, but the nurse kept talking about this. The conversation was led by the preset ideas the nurse had in mind and she didn't ask us: 'Is it all clear?' or 'What kind of information would you like?'. It was like she had to stick to her mission." (Rel.foc1)

"It was a standard story, at least, that was how I experienced it." (Pat.int5)

"I thought what she did, was merely reading aloud the information. What she 'had to do', like 'I just have to tell you this'. (Pat.int4)

In addition, the interviewees stressed the importance of the nurse exploring the *personal situation *of the patient and tailoring information by giving personal (lifestyle) advices.

"They did not ask: 'How's your living condition? Can you amuse yourself?' [..] I think that should be part of it. [..] And maybe they could give advises, like 'you could do this, or that'. You don't hear any of this." (Pat.int4)

"Nothing was asked about my personal situation." (Pat.int5)

Besides the content of information, also the *moment of providing information *depends on individual preferences, as mentioned by the patient groups and interviewees.

"Well, that depends strongly on the patients' preference. Some people say they are really upset when they have just heard the diagnosis, the information just don't get in." (Pat.foc7)

"And then this man, the doctor, said: 'Sir [..], you have cancer'. All I could say was: 'O, o, o.....'. The next thirty minutes I was completely stunned. But then the doctor was going to explain the treatment options. These conversations shouldn't follow directly after one another." (Pat.foc8).

"When she told me the diagnose, the nurse said 'you can go home now and come back [..], we can discuss the treatment options then.' I said: 'Actually I wanted to do this yesterday, in other words: it's already too late. I prefer doing it this afternoon'." (Pat.foc6)

#### Empathy and support

The patient groups and the interviewees stressed the importance of not only tailoring the information to the patients' information needs, but also *showing empathy and support *in order to fulfil the patients' emotional and support needs.

"As a nurse, you need to show empathy to make patients feel comfortable. [..] And psychosocial subjects; I think it's strange these are not discussed." (Pat.foc5)

"Every patient is different, the challenge is to give support in the right manner" (Rel.foc3)

"The way in which she offered it was very reassuring. [..] She was all ears and [..] it was just very human." (Pat.int1)

#### Dispel the patients' fears

Patients mentioned that they experience(d) worries about their health, their treatment and their future. The nurse need to *pay attention to their worries and fears *in order to *dispel *them.

"They [nurses ERP] need to check what a patient is worried about. They [patients ERP] bring up concerns and worries they want to talk about to return home satisfied." (Pat.foc7)

"They try to reassure you, but that's not easy. [..] I was more relieved [after the consultation ERP]. I knew a little bit more about how it would go." (Pat.int5)

Supporting the patient and his relative is not only important during patient education at the beginning of treatment, but for older patients it is essential to provide continuous support *at several moments *during the period(s) of treatment. This is experienced as a gap in current practice.

"Yes, but not just at one single moment. For example, after a few weeks you should create a moment to talk about: 'How are you doing, how did you experience this treatment period, are you able to handle everything..' I consider this important." (Rel.foc4)

"A little chat helps much more than a pill. [..] If I could talk with her, that is much more important than the medical care she has to give. [..] For example: 'How do you feel?' or 'What do you think about this or that?'. That was never asked." (Pat.int3)

#### Patients' expectations and treatment goals

Another aspect in patient education is, according to the professional groups, *exploring patients' expectations or ideas *about treatment with chemotherapy.

"You can try to tailor to the patients' needs by asking: What do you already know? What did the doctor already tell you? What would you like to know more? What are you worried about?" (Res4)

According to the professional groups, the difference in life expectations between older and younger patients can have its impact on *the decision of undergoing a treatment*, such as chemotherapy, as older patients may feel they will not benefit as much from the treatment as younger patients. Therefore, the professional groups stated that the possibility should be created to reflect on the question: do I want to undergo a treatment or not?

"Maybe they [patients ERP] wanted to be asked that question again. This could be asked by the doctor at a later moment, fine, but the possibility should be there to ask this question." (Expert2)

"The alternative (not undergoing a treatment) is often not even discussed." (Expert3)

"Because older people often think they have no choice." (Res6)

According to the professionals and patients groups, an important aspect in the decision of undergoing the treatment is *exploring the goals *patients (and their relatives) have.

"My daughter was pregnant, and when my husband was diagnosed with lung cancer, we immediately said: 'you are going to stay alive', because we wanted to see my grandchild become 20 years old. We absolutely wanted to live for many more years." (Rel.foc4)

"I think it is important to hear what they want to achieve, because that is the drive to undergo the treatment. Some patients explicitly say: 'My daughter is three months pregnant so I want to live at least half a year more." (Nurse3)

#### Addressing patients' relatives

The patient groups especially stressed the importance of nurses *supporting the relatives *as much as the patient itself.

"It's important that there's support for my wife, she's more ill than I am." (Pat.foc3)

"I would like to have the feeling I can contribute in my own way. At a given moment, a nurse explained to me how I could take care of my husband. That was fantastic." (Rel.foc1)

#### Communication training for nurses

According to a patient group and interviewees, nurses' skills to tailor information to the patients' specific needs have to be improved.*Training the nurses *in conversation techniques and specific knowledge about older individuals was considered useful by both patient and professional focus groups. The patient groups additionally pointed out the importance of training the nurse in showing empathy and providing emotional support.

"An important aspect is that nurses receive good training, including knowledge of the difficulties that may exist in older individuals with processing of information." (Expert7)

"Part of the training should be gaining knowledge about older people, but there are more things that should be taught, for example conversation techniques." (Nurse5)

"But that [showing empathy ERP] is not something everybody has, they should be trained to show empathy, so they can focus on patients' needs." (Rel.foc1)

According to the professional group, for a training to be effective it should be completed by a follow-up, in which learning in practice is the key ingredient.

"I think communication training can only result in changed behaviour if you connect it to a follow-up in which learning and working is indeed connected so that working situations become learning situations [..]." (Expert5)

### 3. Enhancing recall of information

#### Bringing a relative

Both patient and professional focus groups mentioned that the patient self can enhance the remembering information by *bringing a relative *to the consultation, because patient and relative both will remember different aspects of the information.

"Bringing someone helps with listening." (Pat.foc5)

"I thought it was pleasant that I brought my children with me. In this way they knew what was going on" (Pat.int3)

"I think children often ask very relevant questions during the consultation, something the father or mother did not think about. Patients often tell more when somebody they know is present. As a nurse, you receive more complete information, like: 'Well mum, you did not yet tell, that...' (Nurse5)

#### Check: does patient understand the information?

For the nurse, there are several ways in which to enhance recall of information by older cancer patients. In the first place, it is important that the nurse *checks to see if the patient understands the information*, according to the patient groups and to one group of professionals (researchers).

"Nurses should ask the patient if there are any questions or if certain things are not clear, both during and after the consultation." (Pat.foc5)

"You should systematically create several moments in which to make sure that everything is clear for the patient." (Res4)

#### Prioritize and offer information in a structured manner

According to the patient and professional groups, nurses have to set priorities about what to address during patient education. Also, they should offer the information in a structured manner and summarize the most important information.

"Prioritize, summarize and repeat it the next time." (Nurse2)

"The whole structure of the conversation: cluster the information and offer the information step-by-step." (Expert5)

The patient and professional groups mentioned that it is helpful for older patients to *receive information *step-by-step, i.e. over different periods of time.

"I would spread the information and check if they remember it. Not too much information at a time." (Res2)

"There should be a possibility for older people to come back another time. Older persons handle emotions in a different manner and they process information more slowly." (Pat.foc5)

#### Speak in clear language

In the patient group, *speaking in plain language *in stead of using technical jargon was mentioned.

"During patient education, they often use technical jargon. They can explain a lot, but in the beginning you're just stunned and nothing gets in. [..] They should explain everything in comprehensible language." (Rel.foc3)

#### Combine different methods of offering information

The professional group, and one interviewee pointed out that it helps to *combine different methods of offering information*, for example written (e.g. booklets), oral, and visual. The patient group did not mention this aspect.

"To combine visual information with oral information, if necessary with handouts so family members can write down information." (Nurse3)

"It's to much to comprehend everything that's why it is useful to receive a booklet. At home you read it over." (Pat.int5)

"But this [combining different methods of information ERP] all involves general information. Even more effective is offering personally relevant information, for example what is discussed during the consultation and what is particularly relevant for this specific patient." (Expert3)

#### Use question list

Finally, both patient and professional groups mentioned the *use of a list with questions or subjects to discuss *during patient education about the treatment.

"The oncology nurse sent me a list with subjects two days before the consultation. This way, I was able to prepare myself. I thought it was pleasant to know what she was going to discuss." (Pat.foc7)

"I think that page in the information folder [e.g. Treatment Guide Chemotherapy ERP] is useful to write down questions to ask the nurse." (Pat.foc3)

"Questions the patient can prepare before the consultation. I think this would yield a profit." (Expert6)

## Discussion

This article describes patients', relatives' and professionals' opinions about older cancer patients' needs preceding cancer treatment, as obtained by five focus group discussions and five one-to-one interviews.

### Tailoring patient education to the patients' needs

All participants mentioned a lack of providing older cancer patients with personally relevant information at the moment they consider appropriate. According to the members of the focus groups, the best way to tailor information to older patients needs is asking patients to articulate these needs. These findings are in line with earlier results [26,27], suggesting that effective communication is enhanced when both parties (e.g. nurse and patient) express their needs. Literature indicates that, compared to younger patients, older patients seem to report less often that they need information about the treatment, how the treatment works and what it accomplishes, what all the possible treatments are, what all the possible side effect are and things you can do to help yourself get well [21]. Furthermore, older patients demonstrate greater reliance on information provided by the physician than younger patients [5]. The results of the present study show that the individual needs of older cancer patients regarding the amount and content of information they require may be insufficiently explored in current practice. Previous studies on patients of all ages also showed that health care professionals seem to have difficulties understanding agendas and needs of their patients [28] and do not appear to explore these needs extensively [29]. The study results suggest that nurses should offer (medical) information the patient himself or herself needs to know in stead of offering information they consider relevant for this patient. One of the problems that have to be solved to be able to provide older cancer patients with tailored medical content information is the lack of evidence regarding costs and benefits of chemotherapy for the older cancer patients. Older people (> 70 years) were largely excluded from early trials on which many of the treatment guidelines are based and when they were included they represented a minority of healthy older people [30-32]. This means that there is still a deficit in knowledge regarding how chemotherapy impacts on older patients in terms of benefits in survival and recurrence offset by costs in terms of side effects and effects on functional status and quality of life. The lack of reliable information that is specific to this age group complicates patient education. Although there are new initiatives to address this gap, additional research is still needed.

The results also show that information needs of older cancer patients as compared to younger patients do not so much differ in the required content of the information, but rather in the way information is given. According to the patient groups, showing empathy and support is an important aspect of patient education. It makes the patient (and relative) feel understood and creates a trustful environment, both during and after the consultation, which is a prerequisite for reflection on the information provided and the decision to undergo treatment. Although showing empathy and support is important in consultations with younger patients too, it could be especially important for older patients and their relatives as the results show that older patients have different life expectations. As a consequence, they might have a different view on the added value of undergoing treatment and perhaps a greater need to deliberate over the decision to undergo the treatment. This finding is in line with earlier research, showing that older cancer patients may be less willing than younger patients to trade increased survival for their quality of live when considering chemotherapy [33]. Therefore, the professional focus groups stated that exploring personal treatment goals can be helpful to explore the patients' wishes surrounding treatment. Salmon and colleagues [34] explained age differences in life expectations by suggesting that older patients may experience fewer cancer-related losses than younger patients due to the age-associated reduction in the time left to live. For example, older patients may feel less disturbed in their future plans than younger patients do. Furthermore, for older patients, their illness may have less impact on their appreciation of life then for younger patients [34]. Research also shows that older adults may have higher levels of psychological resources, such as the ability to adjust their goals to unchangeable circumstances [35]. Besides, older patients seem to need less support in coping with the disease and treatment than younger patients do [21]. Therefore, younger people may need more information from the health care professional in order to cope with their disease and treatment; in contrast, older patients may be overwhelmed by the information [36]. Our results suggest that nurses try to adapt to patients' emotional needs by providing information instead of showing empathy and emotional support, while these latter skills were considered particularly important by the participants in this study.

### Enhancing recall of information

Our findings indicate that especially the way in which information is provided to older cancer patients could be improved. All focus groups stated that to enhance memory in older patients, information should be offered in a structured manner in which the most important, personally relevant information should be summarized and repeated. Furthermore, information should be offered step-by-step, to enable patients to let the information sink in, to consider the personal relevance and make him- or herself familiar with it. This is especially important for older patients, as they often are less educated than younger patient and cognitive functions decrease with age [11,12]. Another key factor that affects comprehension of the information is the language level used to convey the message. Nurses should avoid using jargon that is common to them, but not to older patients. The only way to know if a message is understood and can be recalled is for the nurse to ask patients to repeat the message. Last, combining different methods of offering information (e.g. providing patients with additional written information) might improve the patient's ability to understand and recall the message.

The current study shows that stimulating the patient to bring a (younger) person to the consultation is considered an effective way to express their needs, address specific subjects to discuss and improve remembering. Literature suggests that a supportive accompanying individual may enhance communication when a complicated treatment regimen is being described, if the accompanying individual is present at the patient's request and provides positive support to the patient and accurate information to the health care provider [14,37]. Having the opportunities to receive information and discuss concerns might also be useful for caring relatives to enable them to be more effective in their caring role [38].

### Interventions to implement in practice

To engage patients in patient education and gain insight in their specific information needs, patient and professional focus groups recommended encouraging patient question asking behaviour in cancer consultations. More specifically, a list with questions about different aspects of the treatment can be given to cancer patients before their initial consultation. Such a list is known in cancer literature as a question prompt sheet (QPS) or question prompt list. Patients can mark the questions on the QPS that they want to discuss during consultations [39-41]. Patients who actively participate in consultations by asking questions, are able to change the focus of the consultation, control the duration and the amount of information provided [42] and receive higher levels of information recall [40].

According to both patients and professionals, communication training for nurses could be useful, especially when addressing specific competencies to provide older patients with tailored patient education. The training should contain knowledge about the information processing and memory in older people, but also teach the essential skills of good communication with older cancer patients, such as exploring the patients' information needs and expectations, responding to cues and concerns, showing empathy and emotional support, paying attention to the patients' relatives and guiding the patient in accepting help from others. Research results show mixed results of the effectiveness of communication training for (oncology) nurses. Some researchers found significant improvement of nurses' skills [42,43], others found limited results [44,45]. However, it has been suggested that consolidation follow-up sessions are required to facilitate maintenance of newly acquired skills and their transfer into the clinical practice [46,47].

### Study limitations

The results of this study should be interpreted with care, since only a total number of 38 participants were obtained. In qualitative research, data collection should continue until saturation is achieved, or no new information is uncovered. Usually, three to five group meetings are supposed to be needed to reach this goal [48]. In our study, we organized five meetings and no new items emerged in the last interviews and group discussions, indicating that saturation was achieved. One patient focus group included in this study existed of members of patient associations, probably consisting of active patients, which might have influenced their perspectives about what kind and how much information is relevant to an older patient. To handle this possible bias and include a wider variety of patients, the second group of patients was selected by an advertisement in a common magazine for older people. Moreover, the interviewees were randomly included, based on the date of their second chemotherapy treatment. Although there was an equal number of patients with low education as with high education (see Table 1), some participating patients might still have been verbally more expressive than the average older cancer patient. Besides this, the patient participants were all older cancer patients. Although these patients subjectively compared their current needs and cognitive capabilities to recall information with their own perceived needs at a younger age, we did not ask younger patients about their communication needs preceding treatment. Therefore, we cannot compare the findings of older cancer patients' needs objectively with findings of younger patient's needs. Moreover, the old-old patients were not reached in this study. It is recommended to cover these limitations in future studies. Last, this study only included patients following treatment. Therefore, the reliance is on post treatment recall of patients' pre treatment needs. It can be questioned to what degree patients have explicit (information) needs before treatment, as they are not yet familiar with the medical procedures and the patient education about their treatment. To validate the findings of this research and address possible differences in patients' needs pre and post treatment, it is recommendable to include both patients' pre treatment and post treatment information needs in future studies.

## Conclusion

To summarize, this study gives insight in patients', relatives' and professionals' views on patient education for older cancer patients. The results indicate that patient education should be more tailored to older cancer patients' specific needs, by exploring these needs and responding to them during consultation. Showing empathy and support is an important aspect in patient education of older cancer patients, as older patients have different life expectations. In a trustful environment both during and after the consultation, the possibility can be created to reflect on the information provided and the treatment decision, which is not common in the present setting of patient education.

During consultation, using an information booklet (e.g., the 'Treatment Guide Chemotherapy' in the Netherlands [22]) can function just as a basic guideline and as an aid for patients at home, but the discussion of the contained information should be adapted to and, if necessary, extended to patient's specific information needs. Information should be provided more structured and spread out over different moments, to enhance recall of information by older patients. To engage patients in patient education and gain insight in their specific information needs, older patients' question asking behaviour in cancer consultations should be encouraged. In addition, a communication training for nurses addressing specific competencies to provide older patients with tailored patient education, could be useful.

Future (quantitative) studies are needed to compare older cancer patients' needs to younger ones and to explore the extent to which patients' and professionals' opinions are reflected in daily practice and to develop recommendations for further improvements.

## Competing interests

The authors declare that they have no competing interests.

## Authors' contributions

JCMW and JJ designed the study and constructed the interview protocol. ERP and JCMW categorized the data. ERP drafted the manuscript. JCMW and JMB acquired funding. ERP, JCMW, JJ and JMB haven been involved in critically revising the manuscript. All authors have read and approved the final manuscript.

## Pre-publication history

The pre-publication history for this paper can be accessed here:


